# Role of DEAD-box RNA helicase genes in the growth of *Yersinia pseudotuberculosis* IP32953 under cold, pH, osmotic, ethanol and oxidative stresses

**DOI:** 10.1371/journal.pone.0219422

**Published:** 2019-07-09

**Authors:** Xiaojie Jiang, Riikka Keto-Timonen, Mikael Skurnik, Hannu Korkeala

**Affiliations:** 1 Department of Food Hygiene and Environmental Health, Faculty of Veterinary Medicine, University of Helsinki, Helsinki, Finland; 2 Department of Bacteriology and Immunology, Medicum, and Research Programs Unit, Immunobiology, University of Helsinki, Helsinki, Finland; 3 Division of Clinical Microbiology, HUSLAB, Helsinki University Hospital, Helsinki, Finland; INSERM U1151 - CNRS UMR 8253, FRANCE

## Abstract

*Yersinia pseudotuberculosis* is an important foodborne pathogen threatening modern food safety due to its ability to survive and grow at low temperatures. DEAD-box RNA helicase CsdA has been shown to play an important role in the low-temperature growth of psychrotrophic *Y*. *pseudotuberculosis*. A total of five DEAD-box RNA helicase genes (*rhlB*, *csdA*, *rhlE*, *dbpA*, *srmB*) have been identified in *Y*. *pseudotuberculosis* IP32953. However, their role in various stress conditions used in food production is unclear. We studied the involvement of the DEAD-box RNA helicase-encoding genes in the cold tolerance of *Y*. *pseudotuberculosis* IP32953 using quantitative real-time reverse transcription (RT-qPCR) and mutational analysis. Quantitative RT-PCR revealed that mRNA transcriptional levels of *csdA*, *rhlE*, *dbpA* and *srmB* were significantly higher after cold shock at 3°C compared to non-shocked culture at 28°C, suggesting the involvement of these four genes in cold shock response at the transcriptional level. The deletion of *csdA* ceased growth, while the deletion of *dbpA* or *srmB* significantly impaired growth at 3°C, suggesting the requirement of these three genes in *Y*. *pseudotuberculosis* at low temperatures. Growth of each DEAD-box RNA helicase mutant was also investigated under pH, osmotic, ethanol and oxidative stress conditions. The five helicase-encoding genes did not play major roles in the growth of *Y*. *pseudotuberculosis* IP32953 under pH, osmotic, ethanol or oxidative stress.

## Introduction

*Yersinia pseudotuberculosis* is a psychrotrophic foodborne pathogen that can cause yersiniosis, with symptoms including abdominal pain, fever and post-infectious systematic complications such as reactive arthritis [1]. It can grow at temperature as low as 0°C [2], making it a threat to the safety of foods stored under chilled condition.

Bacteria use a variety of mechanisms to adapt to temperature downshifts. The increase of unsaturated or branched fatty acids in the membrane lipids of *Escherichia coli* [3,4], *Bacillus subtilis* [5] and *Listeria monocytogenes* [6,7] hinders phase separation of cell membrane phospholipids at low temperatures. Moreover, the cold-induced proteins in *E*. *coli*, including the cold shock protein family [8], DEAD-box RNA helicase CsdA [9], exoribonucleases PNPase and RNase R [10], facilitate efficient transcription and translation at low temperatures.

DEAD-box proteins are members in superfamily 2 of the helicase proteins [11,12]. These proteins are important in bacterial RNA metabolism due to their duplex RNA unwinding activity, coupled with ATP hydrolysis or ATP binding [13–15]. They participate in crucial biological processes including mRNA decay, ribosome assembly and translation initiation [15–17]. DEAD-box proteins are characterized by the presence of nine conserved motifs [11]. Despite highly conserved amino acid sequences, the number of DEAD-box protein-encoding genes in the genome sequence varies greatly in various species [18]. *Y*. *pseudotuberculosis* IP32953 is predicted to encode five DEAD-box RNA helicase genes, *yptb0165* (*rhlB*), *yptb0486* (*csdA*), *yptb1214* (*rhlE*), *yptb1652* (*dbpA*) and *yptb2900* (*srmB*) [19]. The importance of *csdA* in the growth of *Y*. *pseudotuberculosis* at 3°C has been shown by Palonen et al. [20] using insertion mutagenesis. Moreover, many DEAD-box proteins are also reportedly involved in the tolerance of other stressors such as heat, pH, oxidative and ethanol stress [21–23]. However, the role of various DEAD-box proteins in the cold tolerance of psychrotrophic *Y*. *pseudotuberculosis* is still unknown, as is whether DEAD-box RNA helicases are also relevant in the tolerance of other stressors. In the present study, we studied the role of five DEAD-box RNA helicase-encoding genes in *Y*. *pseudotuberculosis* under various conditions, including cold, acidic, alkalic, osmotic, ethanol and oxidative stresses.

## Materials and methods

### Bacterial strains and growth conditions

*Y*. *pseudotuberculosis* IP32953 (gratefully received from Elisabeth Carniel, Institut Pateur, Paris, France) was used as the original strain for the construction of all mutant strains (S1 Table). *E*. *coli* DH5α was used for plasmid construction (S1 Table). *Y*. *pseudotuberculosis* IP32953 and plasmid-containing *E*. *coli* DH5α strains were grown in Luria-Bertani (LB) (Lennox, Sigma-Aldrich, St. Louis, MO) broth or on LB agar. *Y*. *pseudotuberculosis* IP32953 was incubated at 28°C. *E*. *coli* DH5α was incubated with shaking at 30°C when expressing temperature-sensitive plasmids pKD4 or pCP20, or at 37°C when expressing pKD46 and pBluescript-*tetR* (hereafter called pBlue-*tetR*).

### Cold shock, RNA isolation and RT-qPCR

**Fig 1:**
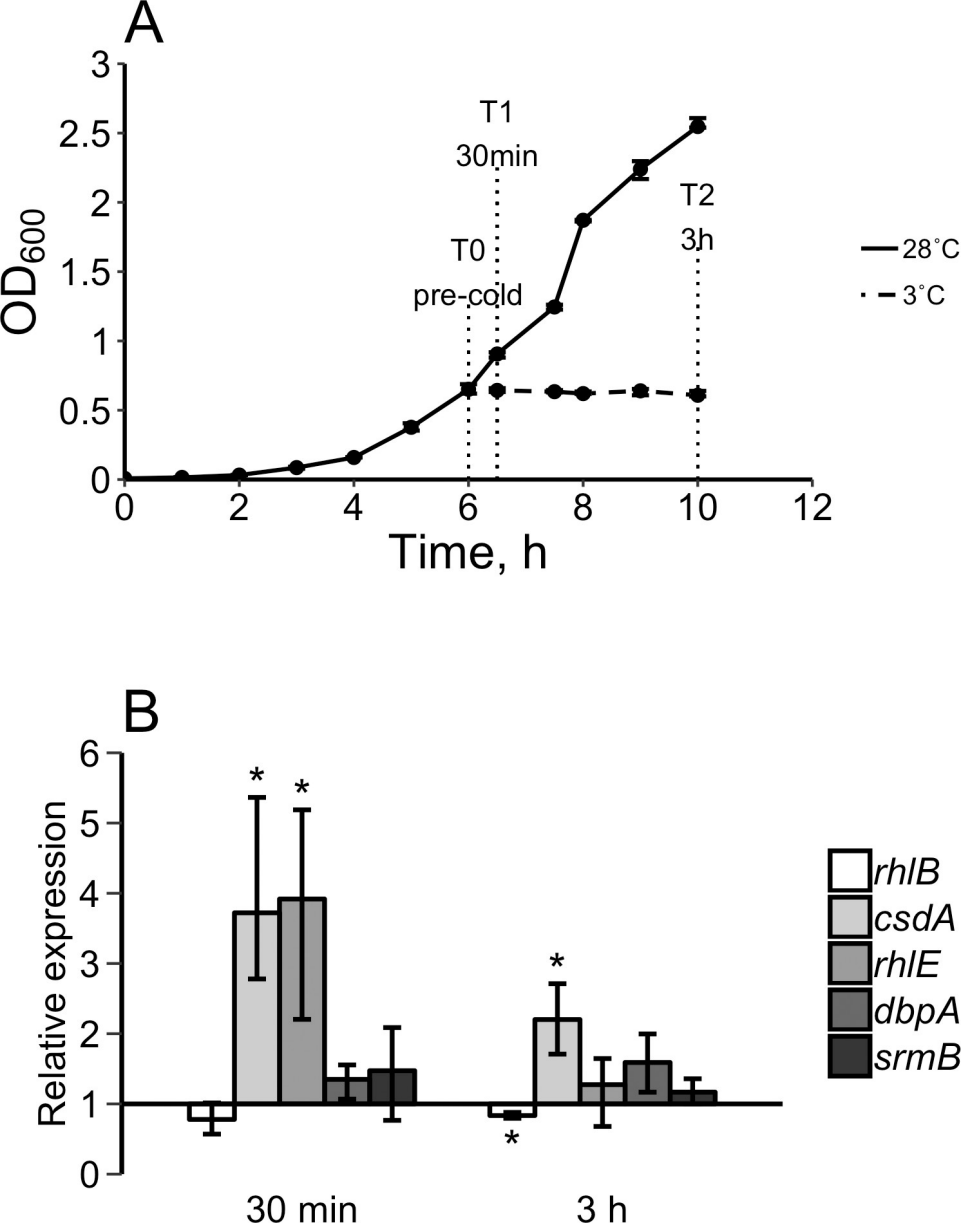
Cold shock of *Yersinia pseudotuberculosis* IP32953. (A) Growth curves of *Y*. *pseudotuberculosis* in cold shock experiment (S2 Table). Three independent colonies of IP32953 were grown at 28°C, exposed to a rapid temperature downshift (cold shock [dash-dotted line]) at 3°C when the optical density at 600 nm (OD_600_) reached 0.5–0.7, and sampled for RT-qPCR analysis before cold shock (T0), and 30 min (T1) and 3 h (T2) after cold shock. Non-shocked cultures (solid line) served as controls. Error bars represent minimum and maximum values. (B) Relative mRNA expression levels of DEAD-box RNA helicase genes *rhlB*, *csdA*, *dbpA*, *rhlE* and *srmB* in three independent cold-shocked (rapid decrease from 28°C to 3°C) cultures compared to pre-cold-shocked cultures at each time point (S3 Table). Significant differences (*P* < 0.05, Student’s t-test) in relative expression levels are indicated with an asterisk. Error bars represent the minimum and maximum values of three biological replicates.

Three replicate cultures were prepared by subculturing of individual colonies of *Y*. *pseudotuberculosis* IP32953 in LB broth at 28°C overnight and diluted (1:100) in fresh LB broth. The diluted cultures were grown at 28°C to the early logarithmic growth phase and the pre-cold-shocked samples (T0) were collected (Fig 1A). One half of each culture was cold-shocked (temperature rapidly decreased from 28°C to 3°C) in an ice-water bath and incubated at 3°C. The other half of each culture (non-shocked culture) was incubated at 28°C. Samples were collected from the cold-shocked cultures at 30 min (T1) and 3 h (T2) after cold shock (Fig 1A). Ten milliliters of each sample was mixed with 2.5 ml of cold stop solution (900 μl of 99.6% ethanol and 100 μl of phenol [Sigma-Aldrich]) and incubated on ice for 30 min. Samples were centrifuged at 2°C 5000 × g for 15 min. The cell pellets were stored at -70°C until RNA isolation.

Total RNA from all samples was isolated using the GeneJET RNA purification kit (Thermo Fisher Scientific, Waltham, MA) followed by DNase digestion using an RNase-free DNA removal kit (Thermo Fisher Scientific) according to manufacturer’s instructions. RNA concentration and purity were measured using the NanoDrop ND-1000 spectrophotometer (Thermo Fisher Scientific). RNA integrity number was measured with an Agilent 2100 Bioanalyzer (Agilent Technologies Inc., Santa Clara, CA) and was at least 9.0 for all samples.

A total of 900 ng of each RNA sample was used to synthesize cDNA using a DyNAmo cDNA Synthesis Kit (Thermo Scientific) for RT-qPCR according to manufacturer’s instructions. The primers for RT-qPCR were designed with Primer-BLAST [24] (S3 Table). The total reaction volume of 20 μl contained 300 ng of random hexamers, 2 μl of M-MuLV RNase H+ reverse transcriptase, 4 μl of template cDNA and a 0.5-μM concentration of each primer. The RT-qPCR was performed with a CFX96 Real-Time Detection System (Bio-Rad Laboratories Inc., Hercules, CA). The cycling protocol of Bio-Rad included initial denaturation at 95°C for seven min, 40 cycles of denaturation at 95°C for 10 s, annealing at 60°C for 15 s, and extension at 72°C for 20 s, with a final extension at 60°C for one min. Fluorescence data were acquired after each extension step. A melt curve after a temperature upshift from 60°C to 98°C (0.5°C / 5 s) was analysed after each run. The amplification reaction efficiencies for each primer pair were defined with a dilution series of pooled cDNA originating from the reverse transcription replicates of the biological replicates. Each dilution was assayed in triplicate. The reaction efficiency was calculated as $10^{\left(-\frac{1}{M}\right)}-1$, where M is the slope of the straight line from a semilogarithmic plot of the quantification cycle (Cq) as a function of the cDNA concentration using Biorad CFX Manager software (Bio-Rad Laboratories Inc.). Reaction efficiencies varied between 0.90 and 1.03.

The relative expression levels of the five helicase-encoding genes were calculated as described earlier by Palonen et al. (2011). Briefly, the expression ratio (R) for each target gene was calculated using equation $R=\frac{{\left(1+E_{target}\right)}^{\Delta C_{q,target}(calibrator-sample)}}{{\left(1+E_{16Srrn}\right)}^{\Delta C_{q,16Srrn}(calibrator-sample)}}$ [25]. In this equation, E_target_ is the amplification efficiency of the DEAD-box RNA helicase-encoding gene transcript, E_16Srrn_ is the amplification reaction efficiency of 16S *rrn* transcripts, ΔC_q,target_ is the C_q_ deviation between calibrator and sample for the DEAD-box RNA helicase-encoding gene transcript and ΔC_q,16S*rrn*_ is the C_q_ deviation between calibrator and sample for the 16S *rrn* transcripts. The cold-shocked samples collected at T1 and T2 were compared to the T0 sample.

### Construction of mutant strains

All five DEAD-box RNA helicase-encoding gene deletion mutants (S1 Table) were constructed as described by Datsenko and Wanner with slight modification [26]. Briefly, a competent *Y*. *pseudotuberculosis* IP32953 wild-type strain was transformed with the λ Red expression plasmid pKD46 (S1 Table). The transformants were grown in ampicillin-containing LB broth, induced with 10% of arabinose and made competent for electroporation as described previously [27]. Linear PCR products were made on the template of kanamycin-resistance cassette flanked by FLP recognition target (FRT) sequences from pKD4 plasmid. The primers (S2 Table) were designed to contain 59 nucleotides from the flanking region of the sequences to be deleted from the IP32953 strain. A 40-°l aliquot of pKD46-containing competent cells was mixed with 400 ng of the linear PCR product in an ice-cold 0.2-cm cuvette (Sigma-Aldrich). Electroporation was performed at 2.5 kV with 25 mF and 200 Ω, immediately followed by addition of 1 ml super optimal broth with catabolite repression medium (Sigma-Aldrich) and incubated at 28°C. The culture was evenly spread on kanamycin-containing LB plates after three hours of incubation. Kanamycin-resistant recombinants were screened by colony PCR with primers k1, k2 and up-/down-stream primers for target genes (S4 Table). The confirmed recombinants were transformed with FLP expression plasmid pCP20 to excise the kanamycin resistance marker (S1 Table) and grown on an LB medium at 37°C to promote curing of the pkD46 and pCP20 plasmids. The final deletion was confirmed by colony PCR with up-/down-stream primers for target genes (S4 Table).

### Complementation of mutant strains

The pBluescript-*tetR* plasmid [28] was used as a vector. The promoter region was predicted by analysing the 500-bp sequence upstream of each DEAD-box RNA helicase target gene with BPROM software (Softberry Inc., Mount Kisco, NY). The coding sequences and native promoters of *dbpA* and *srmB* were amplified by PCR using the primers com yptb1652 NotI, com yptb1652 XhoI, com yptb2900 NotI and com yptb2900 XhoI, respectively (S4 Table). The resulting PCR products and pBlue-*tetR* were digested with *No*tI and *Xho*I (Thermo Fisher Scientific) and ligated with T4 ligase (New England Biolabs, Ipswich, MA), producing pBlue-*tetR*-*dbpA* and pBlue-*tetR*-*srmB*. The complementary plasmid pBlue-*tetR*-*csdA* was constructed by digesting plasmid pBluescript-*csdA* [20] with *Not*I and *Xho*I, and ligated to pBlue-*tetR*.

### Growth experiment and growth curve analysis

Five replicate cultures were prepared by subculturing of individual colonies of *Y*. *pseudotuberculosis* IP32953 wild-type strain, deletion mutant strains and the mutants with complementary plasmids or vectors into 5 ml of LB broth containing 100 μg/ml of ampicillin when appropriate and grown overnight at 28°C. To study the growth at 3°C and 28°C, cultures were diluted into fresh LB broth containing ampicillin when appropriate to OD_600_ of around 0.01.

To study the growth under pH, osmotic, ethanol and oxidative stress, the cultures were diluted into LB broth adjusted to pH 4.6 (with 1 M HCl), pH 4.8 (with 1 M HCl), pH 9.0 (with 1 M NaOH), or containing NaCl (40g/liter) or ethanol (3%, vol/vol) or H_2_O_2_ (10 mM), and ampicillin when appropriate. Biological buffers 2-(N-Morpholino)ethanesulfonic acid hydrate (pH 4.6 and pH 4.8; MES hydrate, Sigma-Aldrich) and N-[Tris(hydroxymethyl)methyl]-3-aminopropanesulfonic acid (pH 8.8; TAPS, Sigma-Aldrich) were used at 200 mM to maintain the desired pH during the growth experiments. The cultures were incubated at 28°C.

A volume of 300°l of each dilution was pipetted into wells of microtiter plates. The microtiter plates were tightly sealed to prevent evaporation during the growth. The strains were grown in the Bioscreen C Microbiology Reader (Growth Curves, Helsinki, Finland). The OD_600_ levels of the cultures were monitored at 15-min intervals at 28°C for 72 h, or at 1-h intervals at 3°C for 720 h. The OD_600_ values were ensured to correspond with the number of viable cells by performing bacterial colony counting using Plate Count Agar plates (Oxoid, Thermo Fisher Scientific) from the wild-type strain and from all the mutants grown at 3°C.

To analyse the defective growth of the deletion mutants, the mean maximum growth rate of each strain at 28°C and 3°C were obtained with DMFit (https://www.combase.cc/index.php/en/), using the model of Baranyi and Roberts [29]. The statistical significances of differences between the fitted maximal growth rates of the mutant strains and the wild-type strain were tested using the Student’s *t* test [30].

## Results

### Relative expression levels of DEAD-box RNA helicase-encoding genes

In the cold-shocked cultures, the relative expression levels of *csdA* and *rhlE* were 3.7- and 3.9-fold upregulated (*P* < 0.05) at 30 min after cold shock compared to T0 cultures, respectively (Fig 1B). The transcripts of *csdA* were also 2.2-fold upregulated at 3 h after cold shock compared to T0 cultures.

### Growth of DEAD-box RNA helicase gene deletion mutants at 3°C and 28°C

**Fig 2:**
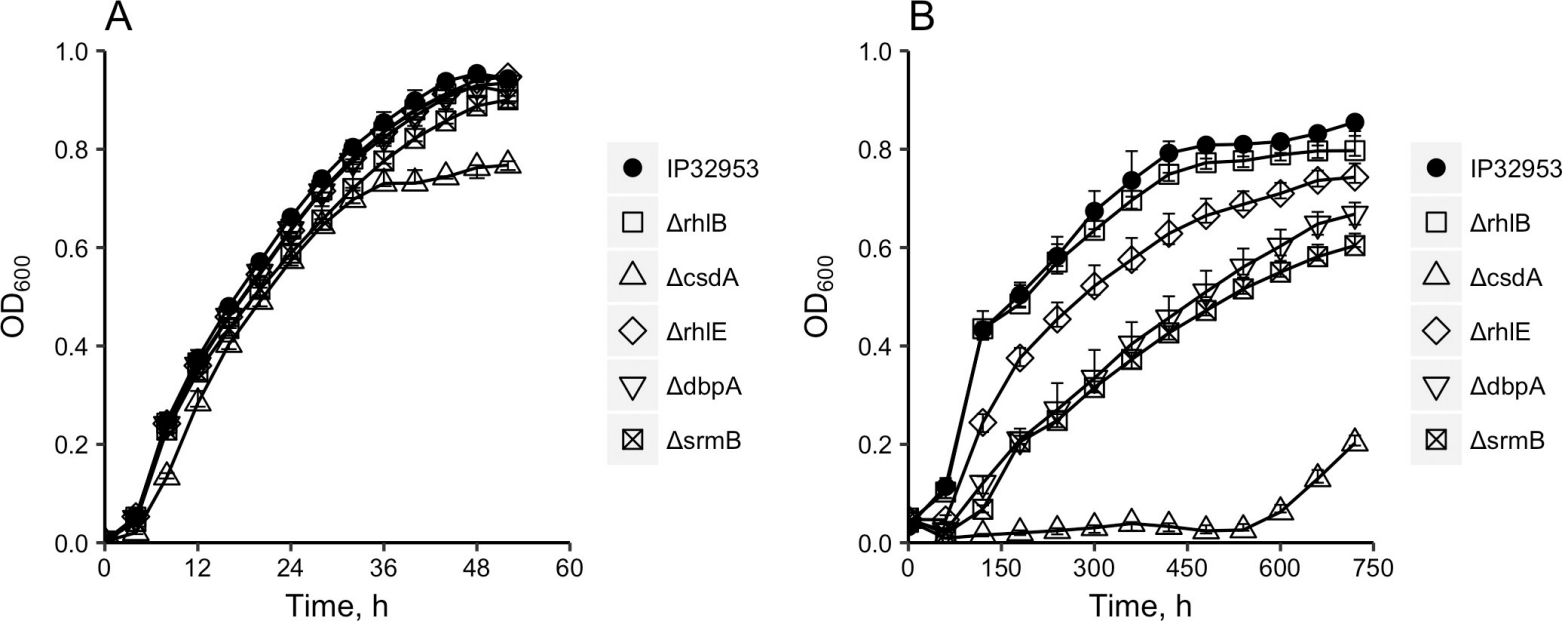
Growth curves of *Yersinia pseudotuberculosis* IP32953 wild-type strain, and mutants Δ*csdA*, Δ*dbpA*, Δ*rhlB*, Δ*rhlE* and Δ*srmB*. All strains were incubated in LB broth at 28°C (A) and 3°C (B). The OD_600_ was monitored at 15-min intervals at 28°C (S5 Table) or at 1-h intervals at 3°C (S6 Table). Points represent the median OD_600_ values of five independent cultures. Error bars represent minimum and maximum values.

Growth of the wild-type *Y*. *pseudotuberculosis* IP32953 and DEAD-box RNA helicase gene deletion mutants Δ*rhlB*, Δ*csdA*, Δ*rhlE*, Δ*dbpA* and Δ*srmB* was monitored at 3°C and 28°C (Fig 2). At 3°C, no observable growth was detected for Δ*csdA* until 600 h of incubation. Mutants Δ*dbpA* and Δ*srmB* showed defective growth compared to the wild-type strain. The maximum growth rate of Δ*dbpA* and Δ*srmB* decreased by at least 50% (*P* < 0.001) compared to the wild-type strain (Table 1). The defective growth of Δ*csdA*, Δ*dbpA* and Δ*srmB* at low temperatures was confirmed with viable cell counting. The viable cell concentration of Δ*csdA* was below the detection limit (200 CFU/ml) after five days of incubation until day 18 at 3°C (Table 2). Three replicates of Δ*csdA* grown at 3°C were inoculated into fresh LB broth at day 18 and incubated at 28°C for 96 h. The viable cell concentration of these cultures reached an average of 7.28 log10 (CFU/ml). Mutants Δ*dbpA* and Δ*srmB* showed significantly lower (*P* < 0.001) colony counts compared to the wild-type strain at day five at 3°C and resumed growth in subsequent time points (Table 2). Mutant Δ*rhlE* displayed a slightly lower growth rate and maximal OD_600_ value compared to the wild type at 3°C (Fig 2B; Table 1). However, the viable cell concentration of Δ*rhlE* did not significantly differ from that of the wild type (Table 2).

**Table 1:** Fitted mean maximum growth rates based on optical density data of *Yersinia pseudotuberculosis* wild-type IP32953 and DEAD-box protein deletion mutant strains at 28°C and 3°C.

Strain	Mean maximum growth rate ± SD (OD_600_ units/h)^*a*^	
	28°C	3°C
WT	0.027 ± 0.0012	0.0024 ± 0.0002
Δ*rhlB*	0.026 ± 0.0005	0.0024 ± 0.0002
Δ*csdA*	0.028 ± 0.0007	0.0012 ± 0.0001 ***
Δ*rhlE*	0.026 ± 0.0007	0.0018 ± 0.0001 ***
Δ*dbpA*	0.026 ± 0.0006	0.0012 ± 0.0001 ***
Δ*srmB*	0.024 ± 0.0011 **	0.0011 ± 0.0001 ***

^*a*^ Significantly decreased values compared to the corresponding value of the wild type are indicated by asterisks (**, *P* < 0.01; and ***, *P* < 0.001) (Student’s *t* test)

**Table 2:** Average viable cell counts of *Yersinia pseudotuberculosis* wild-type IP32953 and DEAD-box protein deletion mutant strains at 3°C after 0, 5, 11 and 18 days.

Time	Viable cell count ± SD [log_10_(CFU/ml)]^*a*^					
(day)	WT	Δ*rhlB*	Δ*csdA*	Δ*rhlE*	Δ*dbpA*	Δ*srmB*
0	4.00 ± 0.031	4.03 ± 0.051	4.09 ± 0.037	3.97 ± 0.064	3.93 ± 0.052	3.97 ± 0.073
5	10.81 ± 0.072	10.64 ± 0.085	-^*b*^	9.88 ± 0.450	8.22 ± 0.467 ***	6.68 ± 0.635 ***
11	10.95 ± 0.099	10.98 ± 0.011	-	10.88 ± 0.094	10.86 ± 0.019	10.84 ± 0.069
18	10.89 ± 0.009	10.95 ± 0.064	-	10.85 ± 0.030	10.81 ± 0.084	10.94 ± 0.044

^*a*^ Significantly decreased values compared to the corresponding value of the wild type are indicated by asterisks (***, *P* < 0.001) (Student’s *t* test)

^*b*^ Below detection limits (200 CFU/ml)

At 28°C, the growth rates of Δ*rhlB*, Δ*rhlE*, Δ*dbpA* and Δ*srmB* were similar with the wild-type strain, and slightly lower maximum optical density compared to the wild-type strain was observed in Δ*csdA* (Fig 2A). The viable cell concentration of these mutants was also similar with that of wild type during growth at 28°C, except a minor difference between Δ*csdA* and wild type after 11 hours incubation (Table 3).

**Table 3:** Average viable cell counts of *Yersinia pseudotuberculosis* wild type IP32953 and DEAD-box protein deletion mutant strains at 28°C after 0, 11, 23 and 28 hours.

Time	Viable cell count ± SD [log_10_(CFU/ml)]^a^					
(hour)	WT	Δ*rhlB*	Δ*csdA*	Δ*rhlE*	Δ*dbpA*	Δ*srmB*
0	3.84 ± 0.122	3.91 ± 0.077	3.84 ± 0.078	3.87 ± 0.064	3.85 ± 0.088	3.84 ± 0.075
11	6.53 ± 0.290	6.69 ± 0.079	5.44 ± 0.179 **	6.72 ± 0.142	6.63 ± 0.127	6.66 ± 0.109
23	9.65 ± 0.043	9.71 ± 0.026	9.72 ± 0.013	9.70 ± 0.062	9.72 ± 0.021	9.74 ± 0.047
28	10.28 ± 0.107	10.20 ± 0.034	10.37 ± 0.081	10.35 ± 0.039	10.21 + 0.078	10.11 ± 0.040

^a^ Significantly decreased values compared to the corresponding value of the wild type are indicated by asterisks (**, *P* < 0.01) (Student’s *t* test)

To verify that the cold-sensitive phenotypes of Δ*csdA*, Δ*dbpA* and Δ*srmB* at 3°C were specifically due to the absence of these genes, the mutant strains were complemented with a wild-type copy of each deleted gene. The complementation restored the growth of Δ*csdA*, Δ*dbpA* and Δ*srmB* (S6 Table; S1 Fig).

### Growth under acidic, alkalic, osmotic, ethanol and oxidative stresses

**Fig 3:**
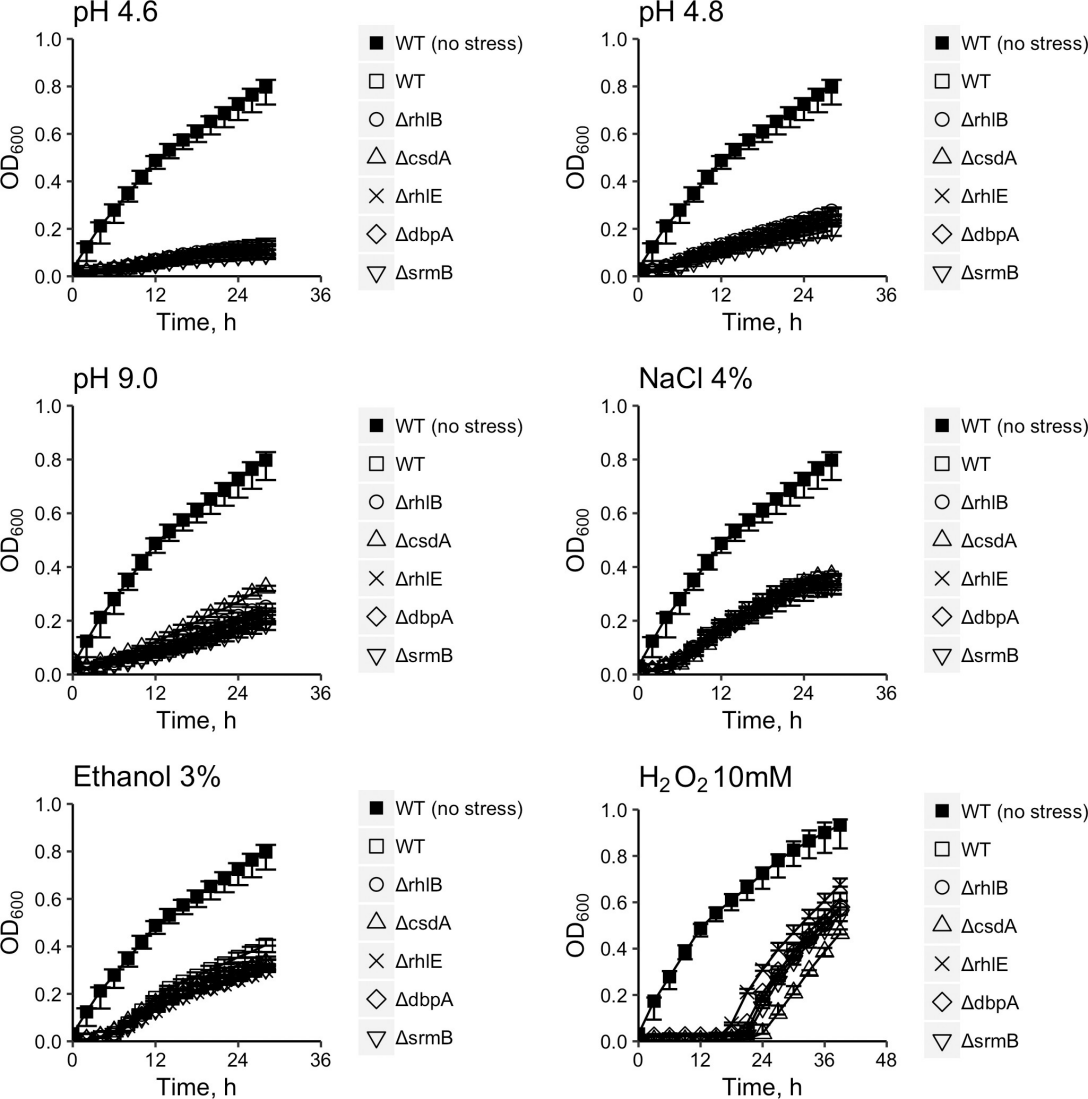
Growth curves of *Yersinia pseudotuberculosis* IP32953 wild-type strain, the DEAD-box RNA helicase gene deletion mutants Δ*rhlB*, Δ*csdA*, Δ*rhlE*, Δ*dbpA* and Δ*srmB* in LB broth at pH 4.6 (with HCl and MES), pH 4.8 (with HCl and MES), or pH 9.0 (with NaOH and TAPS), in NaCl 4%, ethanol 3%, and H_2_O_2_ 10 mM at 28°C. The OD_600_ was monitored at 15-min intervals (S7 Table). The points represent the median OD_600_ values of five independent cultures. Error bars represent minimum and maximum values.

The wild type *Y*. *pseudotuberculosis* IP32953 and DEAD-box RNA helicase gene deletion mutants Δ*rhlB*, Δ*csdA*, Δ*rhlE*, Δ*dbpA* and Δ*srmB* were also incubated and monitored at pH 4.6, pH 4.8 or pH 9.0, in 4% NaCl, in 3% ethanol and in 10 mM H_2_O_2_ (Fig 3). These five mutants showed minor decreases in their growth rates under ethanol stress compared to the wild type (Fig 3; Table 4). None of the five mutants displayed impaired growth rate under pH, salt, and oxidative stresses compared to that of wild type.

**Table 4:** Fitted mean maximum growth rates based on optical density data of *Yersinia pseudotuberculosis* wild-type IP32953 and DEAD-box protein deletion mutant strains without stress and at pH 4.6 (with HCl and MES), pH 4.8 (with HCl and MES) or pH 9.0 (with NaOH and TAPS), in 4% NaCl, in 3% ethanol and in 10 mM H_2_O_2_.

Growth condition	Mean maximum growth rate ± SD (OD_600_ units/h)^a^					
	WT	Δ*rhlB*	Δ*csdA*	Δ*rhlE*	Δ*dbpA*	Δ*srmB*
28°C	0.027 ± 0.0012	0.026 ± 0.0008	0.027 ± 0.0007	0.026 ± 0.0008	0.026 ± 0.0007	0.024 ± 0.0011
PH 4.6	0.004 ± 0.0008	0.005 ± 0.0010	0.005 ± 0.0009	0.005 ± 0.0009	0.004 ± 0.0009	0.004 ± 0.0006
pH 4.8	0.012 ± 0.0013	0.012 ± 0.0003	0.011 ± 0.0005	0.012 ± 0.0007	0.012 ± 0.0006	0.010 ± 0.0008
pH 9.0	0.009 ± 0.0009	0.010 ± 0.0010	0.013 ± 0.0005 **	0.009 ± 0.0010	0.009 ± 0.0013	0.008 ± 0.0011
NaCl 4%	0.018 ± 0.0024	0.018 ± 0.0015	0.019 ± 0.0010	0.018 ± 0.0014	0.018 ± 0.0022	0.015 ± 0.0020
ethanol 3%	0.019 ± 0.0007	0.013 ± 0.0012 **	0.012 ± 0.0005 **	0.012 ± 0.0008 **	0.013 ± 0.0008 **	0.014 ± 0.0010 **
H_2_O_2_ 10mM	0.029 ± 0.0008	0.026 ± 0.0010	0.027 ± 0.0016	0.027 ± 0.0008	0.027 ± 0.0009	0.026 ± 0.0006

^a^ Significantly decreased values compared to the corresponding value of the wild type are indicated by asterisks (**, *P* < 0.001) (Student’s *t* test)

## Discussion

The ceased growth of Δ*csdA* and impaired growth of Δ*dbpA* and Δ*srmB* at 3°C indicate that the DEAD-box RNA helicase-encoding genes *csdA*, *dbpA* and *srmB* are required for optimal growth of *Y*. *pseudotuberculosis* at low temperatures. Gene *rhlE* only played a minor role in low-temperature growth. The growth-ceased *csdA* mutant continued to replicate after moving from 3°C to 28°C indicating that the bacterial cells were still viable. In addition, slight growth was observed for Δ*csdA* after 600 h incubation at 3°C. It is possible that the bacterium is very slowly adapting to low temperature by replacing the deficiency of the csdA by the enhancement of other cold tolerance mechanisms. Previous studies have shown that protein CsdA participates in RNA degradation and ribosome assembly in *E*. *coli* after a temperature downshift [9,31,32]. However, the mechanism of CsdA in bacterial cold tolerance is still unclear. It seems that CsdA is the most important DEAD-box RNA helicase compared to the rest four in the cold tolerance. The distinctive importance of CsdA might stem from its homodimeric form, which is less frequently observed in DEAD-box helicases compared to monomeric form [33], since the dimerization domain of protein CsdA is responsible for its enzymatic function and structural stability [14].

Genes *dbpA* and *srmB* were less important than *csdA* in the cold tolerance of *Y*. *pseudotuberculosis* according to the mutational experiment and transcriptional analysis. In *E*. *coli*, SrmB was required for optimal growth and assembly of the 50S ribosomal subunit at low temperature (22°C) [34,35]. However, deletion of DbpA in *E*. *coli* did not cause a cold sensitive phenotype or inappropriately assembled ribosome at low temperatures (< 30°C) [36,37]. The significance of DbpA may only be observed in psychrotrophic gram-negative strains at refrigerated temperatures. Understanding the mechanisms of the ability of *Y*. *pseudotuberculosis* to grow at low temperatures may provide new tools for controlling this foodborne pathogen.

In our study, gene *rhlE* did not have a notable impact on the cold growth of *Y*. *pseudotuberculosis*. The significant transcriptional response under cold shock stress implies that *rhlE* may function for only a short time after the cell’s exposure to cold stress. Protein RhlE may be a backup helicase for CsdA, as overexpression of RhlE could suppress the cold-sensitive Δ*csdA* in *E*. *coli* [38,39]. However, overexpressed RhlE exacerbates the cold-sensitive phenotype of Δ*srmB* [39]. The helicase RhlE may indirectly participate in cold tolerance by adjusting the activities of CsdA and SrmB.

Gene *rhlB* was not related to the cold tolerance of *Y*. *pseudotuberculosis* according to our mutational or transcriptional analysis. Though RhlB, together with RNase E, PNPase and enolase, was identified as part of the degradosome in *Y*. *pseudotuberculosis* [40], loss of its encoding gene *rhlB* did not harm growth in any stress conditions nor the optimal condition in our study. Further studies are required to confirm whether RhlB is needed for *Y*. *pseudotuberculosis* growth under other untested stresses.

The five DEAD-box RNA helicase-encoding genes are not essential for *Y*. *pseudotuberculosis* growth under acidic, alkalic, osmotic, ethanol or oxidative stresses. All five genes had only minor influence on growth when exposed to ethanol stress. Although DEAD-box proteins have been found to potentially be involved in tolerance to pH, osmotic, ethanol or oxidative stresses in gram-positive *B*. *cereus* and *L*. *monocytogenes* [22,23], they do not appear to play a major role in these stresses in *Y*. *pseudotuberculosis*.

## Conclusion

The helicases CsdA, DbpA and SrmB are important for cold growth of *Y*. *pseudotuberculosis*. CsdA had the strongest impact, with the deletion mutant showing ceased growth at low temperatures. Helicases DbpA and SrmB were less important to cold tolerance compared to CsdA. Moreover, all five helicases only had minor influence on growth under ethanol stress.

## Supporting information

S1 Table Strains and plasmids used in this study. (DOCX)

S2 Table Optical density values (OD_600_) of three replicates of *Yersinia pseudotuberculosis* IP32953 in cold shock experiment for RT-qPCR. (XLSX)

S3 Table Cq (quantification cycle) values, reaction efficiencies of 16S and five DEAD-box helicase genes of *Yersinia pseudotuberculosis* IP32953 in cold shock experiment. (XLSX)

S4 Table Primers used in this study. (DOCX)

S5 Table Optical density values (OD_600_) of growth of *Yersinia pseudotuberculosis* IP32953 and mutants Δ*rhlB*, Δ*csdA*, Δ*rhlE*, Δ*dbpA*, Δ*srmB* at 28°C. (XLSX)

S6 Table Optical density values (OD_600_) of growth of *Yersinia pseudotuberculosis* IP32953, mutants Δ*rhlB*, Δ*csdA*, Δ*rhlE*, Δ*dbpA*, Δ*srmB*, complementation strains Δ*csdA* + pBlue-tetR-*csdA*, Δ*dbpA* + pBlue-tetR-*dbpA*, Δ*srmB* + pBlue-tetR-*srmB*, and vector-control strains Δ*csdA* + pBlue-tetR, Δ*dbpA* + pBlue-tetR, Δ*srmB* + pBlue-tetR at 3°C. (XLSX)

S7 Table Optical density values (OD_600_) of growth of *Yersinia pseudotuberculosis* IP32953 and mutants Δ*rhlB*, Δ*csdA*, Δ*rhlE*, Δ*dbpA*, Δ*srmB* under pH 4.6 (with HCl and MES), pH 4.8 (with HCl and MES), pH 9.0 (with NaOH and TAPS), NaCl 4%, ethanol 3% and H_2_O_2_ 10 mM stresses. (XLSX)

S1 Fig Growth curves of *Yersinia pseudotuberculosis* IP32953 wild-type strain, and mutants Δ*csdA* (A), Δ*dbpA* (B) and Δ*srmB* (C) at 3°C. Data represent growth of wild-type strains (solid circle), deletion mutants (open square), vector-only controls (open triangle) and complemented mutants (open rhombus). The OD_600_ was monitored at 1-h intervals. The points represent the median OD_600_ values of five independent cultures. Error bars represent minimum and maximum values.
(TIFF)
