# Supplementary material for: Role of DEAD-box RNA helicase genes in the growth of Yersinia pseudotuberculosis IP32953 under cold, pH, osmotic, ethanol and oxidative stresses
Source: PLoS One. 2019 Jul 9;14(7):e0219422. doi: 10.1371/journal.pone.0219422 (PMC6615604; doi:10.1371/journal.pone.0219422)
Supplement: S1 Table — (DOCX) [file pone.0219422.s001.docx]

**S1 Table. Strains and plasmids used in this study.**

| Strain or plasmid | Description | Source |
| --- | --- | --- |
| Strains |  |  |
| *Yersinia pseudotuberculosis* | |  |
| IP32953 | Wild-type strain; serotype I | [19] |
| Δ*rhlB* | IP32953-derived mutant, deletion of *yptb0165* (*rhlB*) | This study |
| Δ*csdA* | IP32953-derived mutant, deletion of *yptb0486* (*csdA*) | This study |
| Δ*rhlE* | IP32953-derived mutant, deletion of *yptb1214* (*rhlE*) | This study |
| Δ*dbpA* | IP32953-derived mutant, deletion of *yptb1652* (*dbpA*) | This study |
| Δ*srmB* | IP32953-derived mutant, deletion of *yptb2900* (*srmB*) | This study |
| Δ*dbpAsrmB* | IP32953-derived mutant, deletion of *yptb1652* (*dbpA*) and *yptb2900* (*srmB*) | This study |
|  |  |  |
| *Escherichia coli* |  |  |
| DH5α | Electrocompetent strain | New England Biolabs |
|  |  |  |
| Plasmids |  |  |
| pKD4 | Template for kanamycin-resistance cassette | [26] |
| pKD46 | Red recombinase plasmid | [26] |
| pCP20 | Temperature-sensitive replication and thermal induction of FLP synthesis | [26] |
| pBluescript-*tetR* | Vector for complementation plasmid | [27] |
| pBluescript-*tetR-csdA* | Complementary plasmid encoding CsdA from *Y. pseudotuberculosis* IP32953 | This study |
| pBluescript-*tetR-dbpA* | Complementary plasmid encoding DbpA from *Y. pseudotuberculosis* IP32953 | This study |
| pBluescript-*tetR-srmB* | Complementary plasmid encoding SrmB from *Y. pseudotuberculosis* IP32953 | This study |
