# Supplementary material for: Role of DEAD-box RNA helicase genes in the growth of Yersinia pseudotuberculosis IP32953 under cold, pH, osmotic, ethanol and oxidative stresses
Source: PLoS One. 2019 Jul 9;14(7):e0219422. doi: 10.1371/journal.pone.0219422 (PMC6615604; doi:10.1371/journal.pone.0219422)
Supplement: S4 Table — (DOCX) [file pone.0219422.s004.docx]

**S4 Table. Primers used in this study.**

| Application | Primers | Sequence (5' ⟶ 3')*^a^* | Source |
| --- | --- | --- | --- |
| Mutant strain construction | |  |  |
|  | mut yptb0165 forward | TTATTTCACCGGATACGCTTTCGTAAAGCAATAGTTAGCTGATATTCTACCACACTATGTGTGTAGGCTGGAGCTGCTTC | This study |
|  | mut yptb0165 reverse | GATGGCAGCATAAAGTGAGGTGGAACTTAGCATCATTTATCAGCCTGGTCGCTTACGGTCATATGAATATCCTCCTTAGT | This study |
|  | mut yptb0486 forward | AAATTTTCTCGACGAGTTATGTAGACTGGCTGCCATTATTAATGAGGCACGTGTACATGTGTGTAGGCTGGAGCTGCTTC | This study |
|  | mut yptb0486 reverse | GGTTTTTACGTTTAATCATCAGAGATGACTAAGCGAACTTATGCATCACCGAAGCGACGCATATGAATATCCTCCTTAGT | This study |
|  | mut yptb1214 forward | TGAAACTGCACTTGGCGATATCGTCAGGATTGGGAAGATCTGGAGCTTCTCGTAATATGTGTGTAGGCTGGAGCTGCTTC | This study |
|  | mut yptb1214 reverse | TATCAGTGAATAGAAGCCGTTATACCCAAGAGGTATCGCTTATTCGCCTGGATTACGGCCATATGAATATCCTCCTTAGT | This study |
|  | mut yptb1652 forward | TTGCGCTTGCCTTCAGGCCATCGCTGCCATTCCATCCAGTTATAAGAGTTACTGCCGTGTGTGTAGGCTGGAGCTGCTTC | This study |
|  | mut yptb1652 reverse | ATTTATCGGGATGATGCAACTGCCATCACCCCGCAATGATTATTTTAATAGACGGGCTTCATATGAATATCCTCCTTAGT | This study |
|  | mut yptb2900 forward | CAGGTGAAGAATTCGGCTAACCCGTCTATAATCGGCGCCCCAAGTAGAGGAAGACCATGTGTGTAGGCTGGAGCTGCTTC | This study |
|  | mut yptb2900 reverse | ACCGCTATGTGAGCAGCGGCTTTGTTGTCGTGCATATCACTAACCAGCACTCTTGGTATCATATGAATATCCTCCTTAGT | This study |
|  | |  |  |
| Mutant strain confirmation | |  |  |
|  | mut var yptb0165 U | CCTCGTTGGTGTAGCATTAACC | This study |
|  | mut var yptb0165 D | GGCAGCATAAAGTGAGGTGG | This study |
|  | mut var yptb0486 U | CCAGCAATAGCTGACGAACA |  |
|  | mut var yptb0486 D | CGAGCCCAGTAGCCTGATAG |  |
|  | mut var yptb1214 U | ATGCCTCAATTCATGCCTCG | This study |
|  | mut var yptb1214 D | GCCTCCTAAGAAGCGGTTTT | This study |
|  | mut var yptb1652 U | TCTCTGGTATTATCGCGGCA | This study |
|  | mut var yptb1652 D | ACATCCTTTTCGCTCACAGA | This study |
|  | mut var yptb2900 U | TATAATCGGCGCCCCAAGTA | This study |
|  | mut var yptb2900 D | CGGCTTTGTTGTCGTGCATA | This study |
|  | k1 | CAGTCATAGCCGAATAGCCT | [26] |
|  | k2 | CGGTGCCCTGAATGAACTGC | [26] |
|  | inv-left | TAAGGGTACTATCGCGGCGGA | [39] |
|  | inv-right | CGTGAAATTAACCGTCACACT | [39] |
|  | KvirF-left | TCGTGGCAGCTATGCTGTTC | [39] |
|  | KvirR-right | ATACGTCGCTCGCTTATCCA | [39] |
|  |  |  |  |
| Complementation | |  |  |
|  | com yptb1652 NotI | NNNNNGCGGCCGCGGAGATTACACGTTAATTTGACG | This study |
|  | com yptb1652 XhoI | NNNNNCTCGAGTTATTTTAATAGACGGGCTTTACA | This study |
|  | com yptb2900 NotI | NNNNNGCGGCCGCTCAGTGCCCACTTTCATTGC | This study |
|  | com yptb2900 XhoI | NNNNNCTCGAGCTAACCAGCACTCTTGGTATCAGG | This study |
|  |  |  |  |
| RT-qPCR | |  |  |
|  | RT-yptb0165 F | ACATTCCATTAGTGACCCAC | This study |
|  | RT-yptb0165 R | AATCGCTGGCAAGTTTAATG | This study |
|  | RT-yptb0486 F | GCCGTCTGGATATTCTGATT | This study |
|  | RT-yptb0486 R | ACCGATACGGTGAACATAAG | This study |
|  | RT-yptb1214 F | GCTTAGCCAAGATCAACAAC | This study |
|  | RT-yptb1214 R | ACTCTGTACGTTTTCGTCAA | This study |
|  | RT-yptb1652 F | CTACCCATCAGGTATTGAGC | This study |
|  | RT-yptb1652R | TACAGAACACCACACAAGAG | This study |
|  | RT-yptb2900 F | CCTGCTGCAATACATCAAAG | This study |
|  | RT-yptb2900 R | ATATCTTGGGCAAAACCCAT | This study |

*^a^* N, any of the bases, i.e., adenine (A), cytosine (C), guanine (G) or thymine (T).
