# Supplementary figures and images for: Role of DEAD-box RNA helicase genes in the growth of Yersinia pseudotuberculosis IP32953 under cold, pH, osmotic, ethanol and oxidative stresses
Source: PLoS One. 2019 Jul 9;14(7):e0219422. doi: 10.1371/journal.pone.0219422 (PMC6615604; doi:10.1371/journal.pone.0219422)

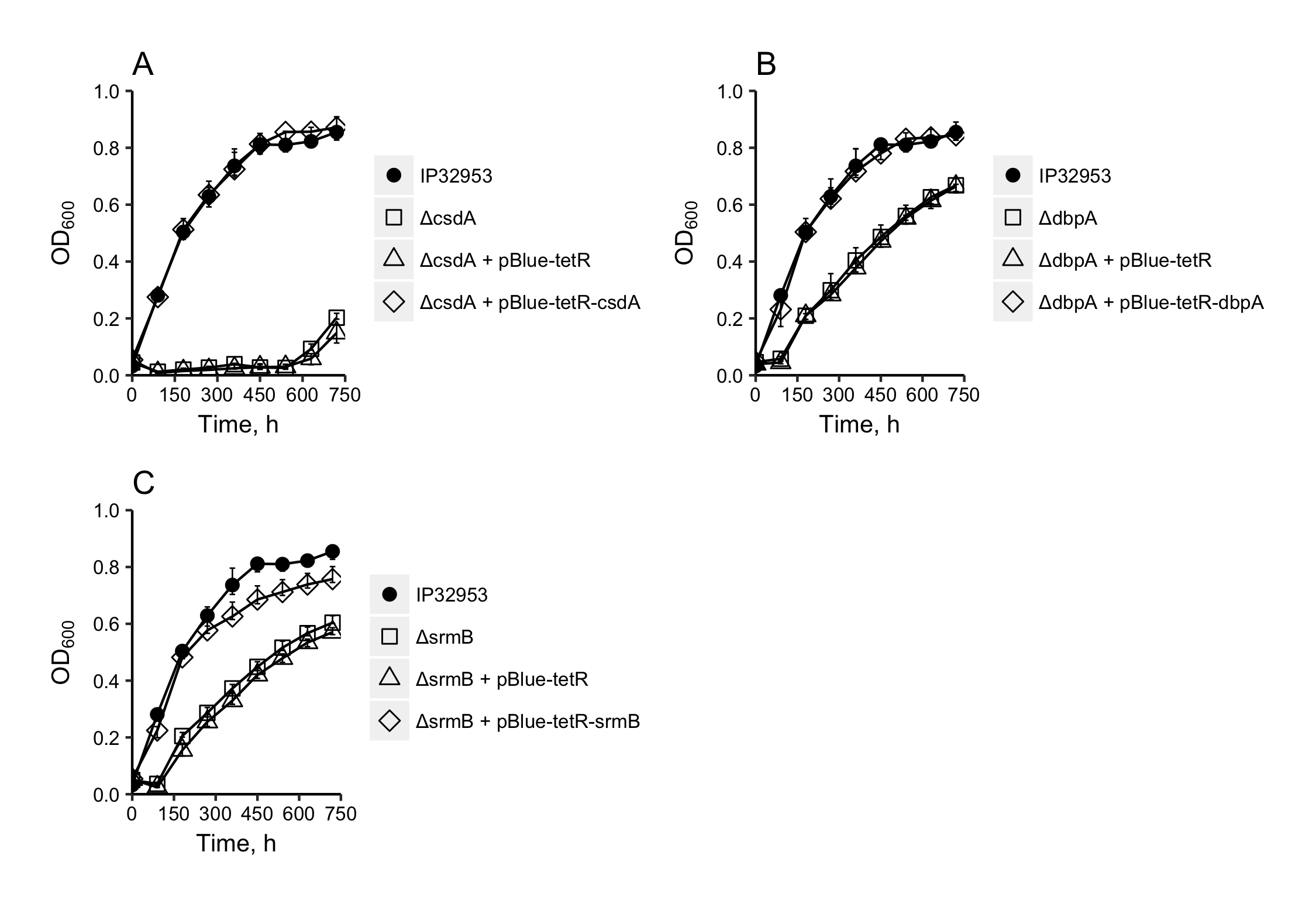

Supplement: S1 Fig — Data represent growth of wild-type strains (solid circle), deletion mutants (open square), vector-only controls (open triangle) and complemented mutants (open rhombus). The OD600 was monitored at 1-h intervals. The points represent the median OD600 values of five independent cultures. Error bars represent minimum and maximum values. (TIFF) [file pone.0219422.s008.TIFF]
